# The role of noncoding genetic variants in cardiomyopathy

**DOI:** 10.3389/fcvm.2023.1116925

**Published:** 2023-05-22

**Authors:** Myo Htet, Shunyao Lei, Sheetal Bajpayi, Asimina Zoitou, Myrsini Chamakioti, Emmanouil Tampakakis

**Affiliations:** ^1^ Department of Medicine, Division of Cardiology, Johns Hopkins University, Baltimore, MD, United States; ^2^ Department of Biomedical Engineering, Johns Hopkins University, Baltimore, MD, United States; ^3^ School of Medicine, University of Patras, Patra, Greece; ^4^ Department of Genetic Medicine, Johns Hopkins University, Baltimore, MD, United States

**Keywords:** cardiomyopathy, noncoding variants, enhancers, promoters, untranslated regions, intronic variants

## Abstract

Cardiomyopathies remain one of the leading causes of morbidity and mortality worldwide. Environmental risk factors and genetic predisposition account for most cardiomyopathy cases. As with all complex diseases, there are significant challenges in the interpretation of the molecular mechanisms underlying cardiomyopathy-associated genetic variants. Given the technical improvements and reduced costs of DNA sequence technologies, an increasing number of patients are now undergoing genetic testing, resulting in a continuously expanding list of novel mutations. However, many patients carry noncoding genetic variants, and although emerging evidence supports their contribution to cardiac disease, their role in cardiomyopathies remains largely understudied. In this review, we summarize published studies reporting on the association of different types of noncoding variants with various types of cardiomyopathies. We focus on variants within transcriptional enhancers, promoters, intronic sites, and untranslated regions that are likely associated with cardiac disease. Given the broad nature of this topic, we provide an overview of studies that are relatively recent and have sufficient evidence to support a significant degree of causality. We believe that more research with additional validation of noncoding genetic variants will provide further mechanistic insights on the development of cardiac disease, and noncoding variants will be increasingly incorporated in future genetic screening tests.

## Introduction

Cardiomyopathies are disorders of the myocardium caused by genetic and environmental factors that eventually result in impaired cardiac function and heart failure (15). Depending on the specific effects in the function and morphology of the heart, and the isolated presence of arrhythmias, cardiomyopathies are divided into dilated, hypertrophic, restrictive, and arrhythmogenic (22). Dilated cardiomyopathy (DCM) is the most common cardiomyopathy affecting 1 in 250 individuals, followed by hypertrophic cardiomyopathy (HCM), which affects 1 in 500, and arrhythmogenic cardiomyopathy (ACM) encountered 1 in 5,000, while the prevalence of restrictive cardiomyopathy is even less common (40). About 30%–50% of cardiomyopathies are heritable, and the different types can have variable phenotypes, prognosis and causal mutations (16, 40, 70, 72, 81). HCM is primarily a disease of the sarcomere, as in up to 60% of patients, a pathogenic or likely pathogenic variant is detected in sarcomeric genes (22, 37). Beta-myosin heavy chain (*MYH7*) and myosin binding protein C3 (*MYBPC3)* are the most frequently affected genes, encoding for proteins of the thick sarcomeric filaments, and patients tend to exhibit disease onset in their forties. Other commonly affected genes in the thin filaments of the sarcomere are cardiac Troponin I (*TNNI3*) and cardiac Troponin T (*TNNT2*) (37). In contrast to HCM, the causative genes in DCM are functionally diverse. Titin (*TTN*) mutations represent 12%–25% of DCM patients and Lamin (*LMNA*) genetic variants represent the second most common mutations in DCM patients (40). Other genes that are associated with DCM are *MYH7*, *TNNT2*, Tropomyosin 1 (*TPM1),* Desmoplakin (*DSP)*, RNA binding motif protein 20 (*RBM20*), and sodium voltage-gated channel alpha subunit 5 (*SCN5A*) (57). In arrhythmogenic cardiomyopathy (ACM), most pathogenic variants are in genes encoding desmosomal proteins such as Plakoglobin (*JUP*) (13, 41), *DSP* (48), Plakophilin-2 (*PKP2*), Desmoglein-2 (*DSG2*) and Desmocollin-2 (*DSC2*) (4, 51, 67). Finally, inherited restrictive cardiomyopathies are caused by mutations in sarcomeric genes such as cardiac troponin I, and less commonly by mutations in Desmin (*DES*) and Filamin C (*FLNC*) (7). It is worth noting that although cardiomyopathies are classified based on phenotypes manifested in the general population, the pathogenic mechanisms and phenotypic features among the various types of cardiomyopathies can overlap to a significant degree.

**Figure 1:**
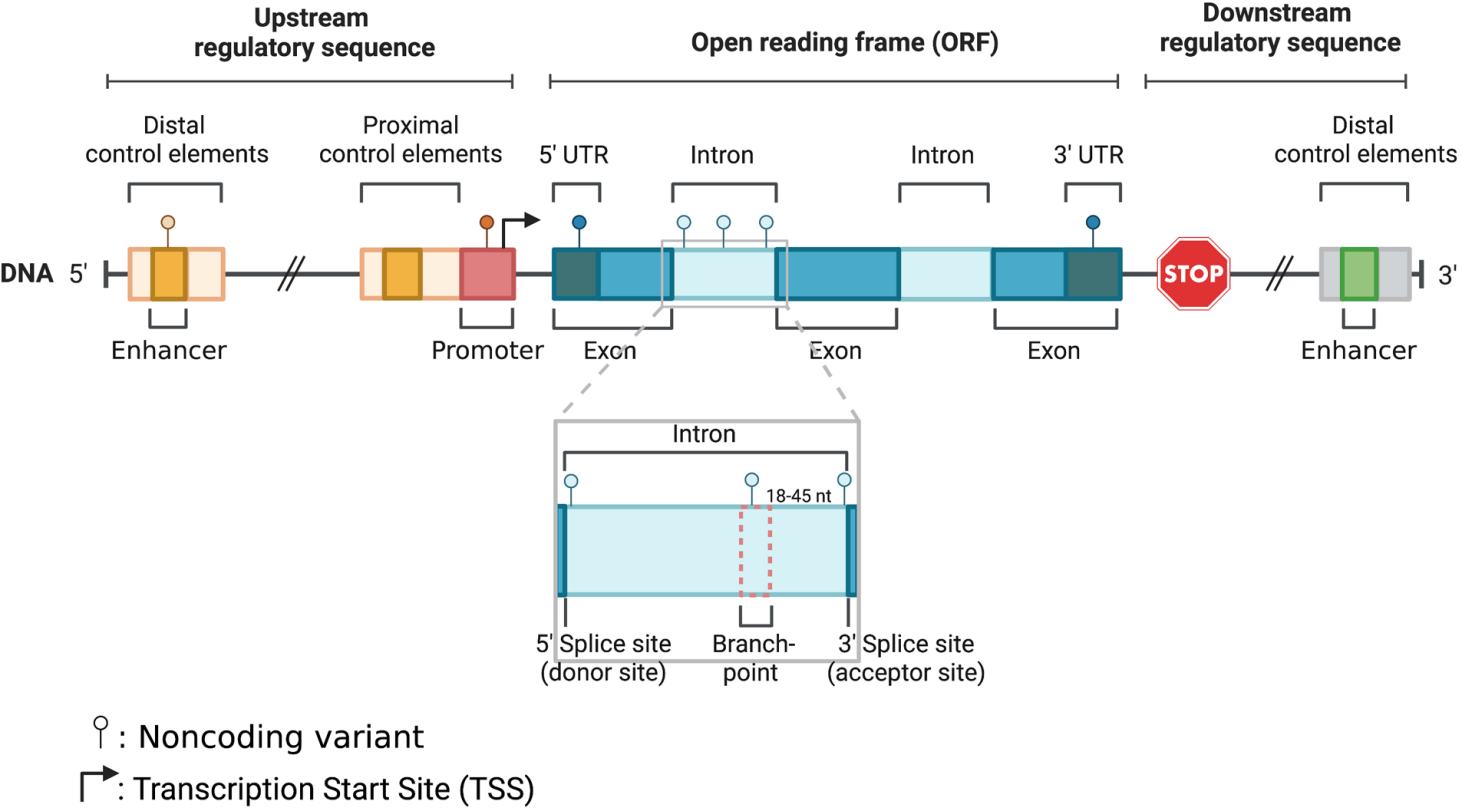
Schematic illustration of noncoding parts in the eukaryotic genome: enhancer, promoter, 5′ UTR, intron, and 3′ UTR.

With the advancement of next generation sequencing and genome wide association studies (GWAS), our understanding of the genetic basis of cardiomyopathies has significantly improved. Multiple GWAS have identified susceptibility loci and variants associated with different types of cardiomyopathies (3, 23, 42, 68, 75). Most rare disease causal variants have been found within the coding region of the genome (81). For example, *TTN* coding variants usually lead to gene truncations and are viewed as the leading genetic causes in DCM patients (25). Contrarily, *MYBPC3* truncating and *MYH7* missense variants are the most pathogenic HCM mutations detected in next generation sequencing research studies (23). Although definitive causative genetic mutations have been identified for familial cardiomyopathies, in over half of the cases targeted genetic screening tests do not identify a contributing variant. This is because most of the current clinical genetic screening tests and earlier research studies relied heavily on whole exome sequencing (WES) or targeted sequencing of coding regions (45, 53, 54). Another explanation regarding the lack of focus in noncoding variants is that even in large meta-analyses, the power of variant detection is limited by variant frequency and penetrance, and lack of systemic interpretation. However, recent evidence from whole genome sequencing (WGS) supports a strong association between genetic variants within noncoding regions and cardiomyopathies (75). Also, emerging evidence corroborates the role of noncoding regulatory regions, where disruption of transcription factor binding sites within enhancers or promoters can alter the 3D chromatin structure and reduce target gene expression, which can be critical for disease (9, 34, 62, 64, 69, 74). Similarly, based on other studies variants within intronic or untranslated regions (UTRs) could also be involved in the pathogenesis of cardiomyopathies (6, 18, 19, 82). Furthermore, according to ClinVar, a significant percentage of non-coding variants in splice sites (∼60%) and UTRs (∼5%), are classified as pathogenic or likely pathogenic (www.ncbi.nlm.nih.gov/clinvar). In this review we will provide an overview of the role of noncoding genetic variants and their association with cardiomyopathies. We will specifically focus on variants within promoter, enhancer, untranslated, splice and intronic regions (Figure 1), where there is sufficient evidence to support a strong association with cardiac disease.

## Cardiomyopathy-associated genetic variants in enhancer and promoter regions

GWAS in heart failure patients have described a strong link with noncoding variants within transcriptional enhancer regions (3, 27, 38, 50, 60, 78) (Table 1). Enhancers are cis-regulatory DNA elements of the noncoding genome that recruit transcription factors to the promoter of target genes for temporal and tissue specific transcription regulation (10, 21, 76). Enhancers play key roles during growth and development, and many studies have shown that disease-associated variants are found within enhancers (52, 56, 74). Furthermore, thousands of cardiac specific enhancers have been described, and it is hypothesized that enhancers may have critical roles in cardiac diseases (1, 14, 24). This was also elegantly illustrated in a recent GWAS, where several regulatory variants in the promoter and enhancer regions were linked to cardiomyopathy (33).

**Table 1:** Variants in enhancer and overlapping promoter regions of genes associated with different types of cardiomyopathies.

Disease	Genomic position (GRCh38)	Enhancer	Promoter overlap	Promoter interaction	Validation method	Proposed pathogenic mechanism	Reference
DCM	Chr14:23438399 (rs875908 C > G)	*MYH7*	–	–	Functional: *in vitro;* phenotype correlation: biobank data	Disrupted TBX-5 binding to *MYH7* enhancer	(20)
DCM	Chr6:144216524C > A	*UTRN*, *STX11*, *SF3B5*	–	–	Case-control analysis; functional: computational tools	Disrupted *UTRN* enhancer function	(12, 73)
DCM	Ch17:75784788 T > C	*UNC13D*, *WBP2*, *SAP30BP* and *TRIM65*	*H3-3B*, *MIR4738* and *UNK*	*TRIM56* and *TMEM94*	Trio analysis	Disrupted interaction with *TMEM94* promoter	(65, 73)
HCM	Chr20:44116250A > G	*JPH2*	–	–	Case-control study	Disrupted *JPH2* enhancer function and altered intracellular Ca^2+^ signaling	(73)
ACM	Chr11:67317729C > T	*GRK2* and *RHOD*	*RAD9A*	–	Trio analysis	Gain of function of *GRK2* enhancer leading to increased *GRK2* kinase activity	(73)
ACM	Chr18:31497935 (-317G > A)		*DSG2*	–	Case-control study, pedigree analysis; functional: *in vitro*	Reduced AP-1 binding to *DSG2* promoter	(11)

DCM, dilated cardiomyopathy; HCM, hypertrophic cardiomyopathy; ACM, arrhythmogenic cardiomyopathy.

### Dilated cardiomyopathy

A study by Gacita et al. demonstrated a potential association between a variant upstream of the *MYH7* enhancer (rs875908) and DCM (20). Genetic deletion of this region in human induced pluripotent stem cell-derived cardiomyocytes (hiPSC-CMs) reduced *MYH7* expression and increased the alpha to beta myosin heavy chain ratio. It is predicted that this region is bound by the transcription factors GATA4 and T-box transcription factor 5 (TBX5) and that this variant likely disrupts the TBX5 binding motif. Interestingly, data from the US Northwestern biobank revealed that the same variant is associated with cardiac function in patients with heart failure. The authors also identified more than 1,700 putative enhancer variants in genes important for cardiac function such as *TNNT2*, Natriuretic peptide A (*NPPA)*, Gap junction protein alpha 5 (*GJA5*) and Myocyte enhancer factor 2A (*MEF2A*) etc. (20). Recently, a study led by Vadgama and colleagues analyzed WGS data of 143 parent-offspring trios from the Genomics England 100,000 Genomes project, and found novel noncoding *de novo* variants in enhancer and promoter regions associated with cardiomyopathy (73). Furthermore, this study reported on a DCM patient who harbored a variant within an enhancer region which was predicted to regulate multiple genes such as Utrophin (*UTRN*), Syntaxin 11 (*STX11)*, and Splicing factor 3B subunit 5 (*SF3B5*). Indeed, animal studies have shown that *UTRN* deficient mice develop DCM (12). Curiously, another DCM patient from the same cohort harbored a variant in an enhancer region that regulates multiple genes such as Unc-13 homolog D (*UNC13D*), WW domain binding protein 2 (*WBP2*), SAP30 binding protein (*SAP30BP*) and Tripartite motif containing 65 (*TRIM65*). Importantly, this enhancer region interacts with the distal promoter region of Transmembrane protein 94 (*TMEM94*), and biallelic *TMEM94* truncating mutation is associated with congenital heart defects (65).

### Hypertrophic cardiomyopathy

In the study by Vadgama et al. one HCM patient was reported to carry a variant within the enhancer of the junctophilin-2 gene (*JPH2*) (73). Junctophilin-2 is a major structural protein in cardiomyocytes, where it also plays a critical role in calcium handling. Heart failure is commonly associated with downregulation of *JPH2,* and mutations in *JPH2* can result in HCM (55, 65, 77). Thus, it is possible that disrupted *JPH2* can alter cytoplasmic calcium signaling leading to cardiomyopathy.

### Arrhythmogenic cardiomyopathy

ACM was linked with a variant within the enhancer of G protein coupled receptor kinase 2 (*GRK2*) and Ras homology family member D (*RHOD*) (73). Furthermore, it was shown that this enhancer region overlaps with the promoter of RAD9 checkpoint clamp component A (*RAD9A*). Importantly, GRK2 expression is upregulated in heart failure and GRK2 inhibition improves cardiac remodeling (59). Recently, a rare noncoding variant (DSG2-317G > A) in the *DSG2* promoter, was also associated with ACM. This heterozygous variant segregated in two daughters of the proband and experimental validation showed a disrupted binding site for the transcription factor AP-1 (11).

## Cardiomyopathy-associated genetic variants in untranslated regions

5′ and 3′ UTRs are key mediators of post-transcriptional gene regulation. They impact mRNA processing, localization and stability (30, 44, 61). They also regulate downstream translation through elements including upstream open reading frames (ORFs), internal ribosome entry sites (IRES), m7G cap, polyadenylation signals, microRNA binding sites and secondary structures (26, 30, 39, 44). With WGS and the advancement of global RNA structure probing *in vivo* (46), an increasing number of UTR variants have been discovered and studied for their association with diseases (32, 63, 80) (Table 2). In addition, several UTR variants appear to increase the risk for disease because of differential microRNA binding affinity to altered alleles and subsequent changes in gene expression regulation (47, 66). Interestingly, more than 45,000 microRNA binding sites in 3′ UTRs of human genes had been discovered by 2009 (17), and these regulate nearly half of the transcriptome (58).

**Table 2:** Variants in 5′ and 3′ UTR of genes that are associated with different types of cardiomyopathies.

Disease	Variant location	Gene regulated	Validation method	Proposed pathogenic mechanism	Reference
DCM	(TATC)2 and (TATC)2/(CAA)2 in 3′ UTR	*RTN4*	Case-control study;	TATC insertion and altered Nogo isoform expression	(82)
DCM	3′ UTR (rs6489956 C > T)	*TBX5*	Case-control study; functional: *in vitro* and *in vivo*	Increased miR9 and miR30a mediated downregulation of *TBX5*	(79)
ACM	5′ UTR (rs770828281 −36G > A)	*TGFβ3*	Case control study; functional: *in vitro*	Loss of auto-inhibitory truncated *TGFβ3* isoform	(6)
ACM	3′ UTR (1723C→T)	*TGFβ3*	Case control study; functional: *in vitro*	Unknown, likely involved altered miRNA mediated regulation	(6)

DCM, dilated cardiomyopathy; ACM, arrhythmogenic cardiomyopathy.

### Dilated cardiomyopathy

In a study of 159 DCM patients and 215 control subjects, Zhou et al. showed an association of DCM with TATC and CAA insertion/deletion polymorphisms in 3′ UTR of Reticulon 4 (*RTN4*) gene (82). The gene codes for NOGO isoforms that have been previously linked with heart failure. (TATC)2 allele and (TATC)2/(TATC)2 genotypes were reported to be associated with an increased risk for DCM. However, there are still limited insights on the functional role of this mutation as it could not be matched with any known 3′ UTR functional motifs. In the Han Chinese population, a 3′ UTR variant in the *TBX5* gene, increased the risk for congenital heart disease such as atrial and ventricular septal defects by nearly two-fold (79). The mutant allele has increased binding affinity to microRNAs-9 and 30a which decreases *TBX5* expression.

CTG repeat expansion in the 3′ UTR of the myotonic dystrophy protein kinase (*DMPK*) gene has been linked to myotonic dystrophy type 1, a neuromuscular disease that can cause cardiac conduction disorders and cardiomyopathy (8). Transcription of this expansion results in CUG repeats that fold into hairpin loops, and sequestration of the nuclear protein muscleblind like (*MBNL*) and heterogeneous nuclear riboprotein 1 (*hnRNPH1*) splicing regulators leading to aberrant alternative splicing of numerous pre-mRNAs (35).

### Arrhythmogenic cardiomyopathy

Through targeted genomic DNA sequencing in a small cohort in ACM patients, Beffagna et al. described two mutations in the 5′ and 3′ UTR regions of the transforming growth factor-beta3 (*TGFβ3*) gene (6). They reported a 5′UTR variant (c.−36G > A) in all clinically affected individuals of the family and in 3 asymptomatic relatives. *TGFβ3* has 11 upstream open reading frames (uORFs). ATG at position -142 translates to a truncated 88 amino acid peptide that has been shown to inhibit the translation of full length *TGFβ3* (2)*.* It was also hypothesized that the 5′UTR variant may result in loss of function of the inhibitory truncated peptide isoform leading to increased *TGFβ* signaling and fibrosis. The disease mechanism for the 3′ UTR mutation found in one patient with ACM, has not been well studied (6).

## Cardiomyopathy-associated genetic variants at deep intronic sites

Deep intronic variants are defined as those located more than 20 bp away from exons, and function to introduce aberrant splicing, distort transcription regulatory motifs, alter non-coding RNA activities, etc. (31). The identification and interpretation of these variants remains challenging due to the large size of introns and lack of consensus sequences (Table 3).

**Table 3:** Deep-intronic variants related to different cardiomyopathies and their proposed pathogenic mechanism.

Disease	Genomic position	Affected gene	Validation method	Proposed pathogenic mechanism	Reference
HCM	c.499+367T > C	*VCL*	Pedigree analysis; functional: computational tools	Disruption of transcriptional motif bindings	(43)
HCM	c.1234−317T > G	*PRKAG2*	Pedigree analysis; functional: computational tools	Disruption of transcriptional motif bindings	(43)
HCM	c.1224−52G > A, c.1224−80G > A, c.1224−21A > G, c.906−36G > A, c.1898−23A > G, c.1090+453C > T, c.1091−575A > C, c.1928−569G > T, c.3331−26T > G	*MYBPC3*	Pedigree analysis; functional: *in vitro*, computational tools	Cryptic splice site, branchpoint disruption and intron retention, leading to haploinsufficiency	(5, 28, 36, 71)

All variants listed according to their reported sequence. HCM, hypertrophic cardiomyopathy; ACM, arrhythmogenic cardiomyopathy.

### Hypertrophic cardiomyopathy

Variants found in deep intronic regions of Vinculin (*VCL*) and protein kinase AMP-activated non-catalytic subunit gamma 2 (*PRKAG2*) were associated with HCM (43). In this study, both *VCL* (c.499 + 367T > C) and *PRKAG2* variants (c.1234−317T > G) are predicted to be deleterious based on computational algorithms, and have a higher prevalence in patients with cardiomyopathy compared to the general population. Additionally, it has been demonstrated through pedigree analysis that the splice site mutation in *MYBPC3* needs to co-exist with the *VCL* variant for disease manifestation. Moreover, these deep intronic variants appear to be enriched in binding sites for specific transcription factors such as FOS, JUN and EP300, and thus they may disturb the transcriptional regulation of cardiomyocytes.

As predicted by comprehensive computational analyses (SpliceAI - prediction tool for cryptic sites, and LabBranchoR - prediction tool for branch point at the splice site (49), the study found *MYBPC3* harbors four splicing-site variants (three in intron 13: c.1224−52G > A, c.1224−80G > A, and c.1224−21A > G; one in intron 9: c.906−36G > A) which result in cryptic splice sites, while one variant (c.1898v23A > G) is likely disrupting a branchpoint in intron 19 and results in nonsense mediated decay-led haploinsufficiency in HCM patients (36). Moreover, an earlier report in two South Asian HCM cohorts revealed a rare pathogenic intronic *MYBPC3* variant (c.1224−52G > A) where the mutation introduces a cryptic splice acceptor site in intron 13 and 50 nucleotide inclusion, which led to altered reading frame and premature termination codon at position 438 (p.Ser408fs*31) (23). Furthermore, a different study in a French HCM patient cohort suggested that deep intronic pathogenic *MYBPC3* variants account for about 6% of HCM highlighting the need for routine *MYBPC3* intronic NGS (29). Moreover, WGS and transcriptomic analysis identified three other *MYBPC3* deep intronic variants (c.1090 + 453C > T, c.1091−575A > C, c.1928−569G > T) in HCM patients (5, 28). RNA analyses were performed to confirm aberrant splicing through the inclusion of cryptic exons in cardiomyocytes from patient-derived induced pluripotent stem cells (iPSC-CMs) and in a myectomy sample from one affected relative of the proband (c.1928−569G > T only). In addition, the role of one *MYBPC3* intronic variant (c.3331−26T > G) was found to account for a genotype-negative proband in a family with a history of HCM (71). This variant segregates with two diseased descendants of the proband and it was found in unrelated HCM patients. Through splicing assays using minigene and patient's blood, the authors confirmed that the variant leads to the retention of intron 30 and thus protein haploinsufficiency.

## Conclusions

Over the last two decades, numerous novel genetic variants have been linked with different types of cardiomyopathies. However, with more information comes greater responsibility, and given the variable penetrance of genetic mutations and lack of in-depth validation studies, attributing causality to most genetic variants has been challenging. Unsurprisingly, this becomes even more complicated when assessing noncoding genetic variants. Nevertheless, analysis of noncoding variants has witnessed tremendous advancements in sequencing techniques and the booming of artificial intelligence. The transition of common methodologies from traditional WES and pedigree analysis to more advanced sequencing incorporated with *in silico* studies and prediction algorithms, fuels the discovery of *de novo* noncoding variants with a potential disease-causing or modifying role in cardiomyopathies. In this review, we provided an overview of the progress in uncovering noncoding variants and their potential pathogenic mechanisms linked with different cardiomyopathies. Given the accumulation of more genetic information and computational tools, the role of some noncoding variants in key genes can be explored further leading to a better understanding of cardiomyopathy mechanisms. Additionally, it is now more obvious that further validation of noncoding genetic variants is missing. Both *in silico* analyses and prediction tools are limited by the population base of rare cardiomyopathies and the oversimplification of disease mechanisms, which result in discrepancies and inaccurate classifications. This supports the development and optimization of more research protocols such as standardized high-throughput *in vitro* testing platforms. Moreover, patient-derived iPSCs serve as an invaluable tool in studying or modeling disease mechanisms and thus could be exploited to functionally annotate and validate the causal roles of certain noncoding variants. In addition, the rapidly evolving field of gene editing with CRISPR technologies, would further accelerate the deeper interrogation of non-coding genetic variants.

Protein coding genes comprise only a small percentage of the entire human genome and frequently their mutations cannot fully account for the observed clinical phenotypes. Noncoding genetic variants have been previously overlooked and it is gradually becoming more obvious that they have more meaningful contributions to cardiac diseases. Therefore, incorporating noncoding variants in genetic screening and demonstrating a potential association with clinical prognosis is foreseeable and could be established as part of personalized medicine in the near future.
